# Efficient materially nonlinear $\mu$FE solver for simulations of trabecular bone failure

**DOI:** 10.1007/s10237-019-01254-x

**Published:** 2019-11-20

**Authors:** Monika Stipsitz, Philippe K. Zysset, Dieter H. Pahr

**Affiliations:** 1 Institute of Lightweight Design and Structural Biomechanics, TU Wien, Vienna, Austria; 2 ARTORG Center for Biomedical Engineering Research, University of Bern, Bern, Switzerland; 3 Division Biomechanics, Karl Landsteiner University, Krems, Austria

**Keywords:** Nonlinear material, Micro finite element, Trabecular bone, Yield strength

## Abstract

An efficient solver for large-scale linear $\mu \hbox {FE}$ simulations was extended for nonlinear material behavior. The material model included damage-based tissue degradation and fracture. The new framework was applied to 20 trabecular biopsies with a mesh resolution of ${36}\,{{\upmu }\hbox {m}}$. Suitable material parameters were identified based on two biopsies by comparison with axial tension and compression experiments. The good parallel performance and low memory footprint of the solver were preserved. Excellent correlation of the maximum apparent stress was found between simulations and experiments ($R^2 > 0.97$). The development of local damage regions was observable due to the nonlinear nature of the simulations. A novel elasticity limit was proposed based on the local damage information. The elasticity limit was found to be lower than the 0.2% yield point. Systematic differences in the yield behavior of biopsies under apparent compression and tension loading were observed. This indicates that damage distributions could lead to more insight into the failure mechanisms of trabecular bone.

## Introduction

In-silico modeling of bone can help get a better insight into the biomechanical behavior of bone (Keaveny et al. 2001). Especially bone failure is not yet well understood, due to the highly complex hierarchical composition of bone. Understanding bone failure could aid in reducing bone fractures due to better diagnostics or in the development of improved treatments. Simulations based on computed tomography (CT) scans provide information on the internal failure progression of bone under loading in more detail than which is currently possible with experiments. The development of high-resolution $\mu$CT scanners made simulations on real bone structures possible. Scan resolutions are high enough to uncover local damage patterns on the micro-scale, i.e., on the level of single trabeculae. Thus, seen as a complementary approach to experiments, simulations can aid in unveiling the invisible failure processes within bones.

Two different modeling approaches for bone structures are commonly used: homogenized, continuum-level methods, and high-resolution microstructural models (Engelke et al. 2013). Homogenized models are based on coarse meshes which do not resolve the trabecular network. Instead, the internal substructure is usually taken into account via density-dependent material laws. Complex material models can be applied at the homogenized material point, due to the small model sizes. In contrast, $\mu \hbox {FE}$ analyses are performed at the microstructural level where the trabecular network is visible. Huge model sizes lead to high computational demands. Thus, only relatively simple material models are feasible. The high resolution of $\mu \hbox {FE}$ models leads to detailed results while keeping the modeling effort low (van Rietbergen and Ito 2015) when compared to hFE.

The challenge of using nonlinear $\mu \hbox {FE}$ analyses in basic research consists of two parts (Nawathe et al. 2014): (1) Whole bones at sufficiently high resolutions need to be simulated. If smaller regions of interest are chosen, results may depend strongly on the actual segment (Mueller et al. 2011) and the chosen boundary conditions (Panyasantisuk et al. 2016). For reliable results, voxel sizes need to be smaller than a third of the mean trabecular diameter, typically around ${40}\,{\upmu \hbox {m}}$ (Bevill and Keaveny 2009). This leads to huge model sizes. (2) A material model that captures the main features of tissue-level failure is required (Nawathe et al. 2014). Thus, $\mu \hbox {FE}$ simulations are always a tradeoff between the computational demands and the complexity of the material model.

Different $\mu \hbox {FE}$ solvers were applied in the literature depending on the model size: smaller $\mu \hbox {FE}$ models (up to a few million degrees of freedom (mio DOF)) are usually solved with commercial or in-house software packages. These solvers are often general-purpose FE tools that are not very efficient (Wolfram et al. 2012; Hambli 2013; Harrison et al. 2013; Baumann et al. 2016; Verhulp et al. 2008). Larger models are commonly solved using specialized research software based on a linear-elastic constitutive law. A number of highly parallel HPC solvers were developed which were able to process models containing hundreds of millions of elements (Adams et al. 2004; Flaig and Arbenz 2012; Mueller et al. 2011). A few of these codes were extended for the use of nonlinear material models at high resolutions. Simulations with more than 200 mio elements were presented (Fields et al. 2012; Christen 2012; Nawathe et al. 2014; Zhou et al. 2016). However, these solvers have not been able to establish themselves in the community, probably due to the high computational demands. For instance, for a model consisting of 120 mio elements, over 4000 CPUs and 120 TB of memory were required (Nawathe et al. 2014). So although it has been proven that nonlinear simulations of whole bones are possible, there is still no nonlinear $\mu \hbox {FE}$ solver capable of analyzing large-scale models on standard HPC clusters.

Another challenge is that a nonlinear material model is required for the investigation of the failure mechanisms of bone. There is no agreement on what features have to be included to accurately model bone failure: in commercial FE packages, simplified micro-level material models are available, where the nonlinearity often consists of a bilinear form in maximum principal stress (Niebur et al. 2000; Verhulp et al. 2008) or a cast iron model (Wolfram et al. 2012). Special user-defined material laws were developed which use, e.g., a quadric yield surface (Schwiedrzik et al. 2013) or a modified von Mises criterion combined with ideal plasticity (Sanyal et al. 2012; Nawathe et al. 2013). In the large-scale simulations, typically no softening mechanisms are present. Thus, the failure behavior cannot be studied directly. Only one large-scale study including tissue failure exists. In this study, bone was modeled as a fully brittle tissue (Nawathe et al. 2015). However, an efficient large-scale $\mu \hbox {FE}$ solver incorporating effects beyond the yield limit is still missing.

The aim of this work is to develop such a nonlinear $\mu \hbox {FE}$ solver for large-scale applications. We follow two main objectives:

1. An existing $\mu \hbox {FE}$ solver is extended to a damage-based material model including a fracture mechanism. We start from ParOSol (Flaig and Arbenz 2012) which was shown to efficiently perform linear analyses on whole bones. By carefully adapting ParOSol to a simple nonlinear material behavior, we expect that the excellent performance can be preserved.
2. The potential of the new solver for biomechanical applications is demonstrated by studying the axial failure behavior of trabecular bone biopsies. The possible areas of application for the high level of detail obtained in the results are investigated.

## Materials and methods

For objective (1), ParOSol (Flaig 2012) was extended to nonlinear material behavior. ParOSol was chosen because it is a highly parallel, efficient $\mu \hbox {FE}$ solver (Flaig and Arbenz 2012). It has a much lower memory footprint compared to standard $\mu \hbox {FE}$ solvers (Flaig and Arbenz 2011). The linear equations are solved by a preconditioned conjugate gradient algorithm based on a geometric multigrid preconditioner. The mesh is stored in an octree. However, only a linear-elastic constitutive law was included in the original ParOSol. A simple material model was required to extend the solver without loosing its good parallel performance.

### Material model

**Fig. 1:**
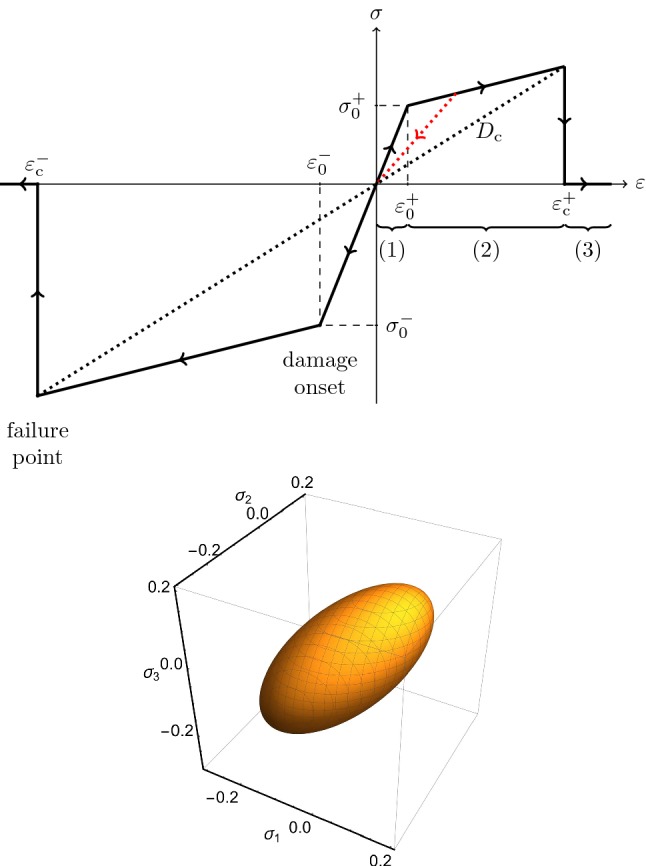
Top: one-dimensional material model showing the (1) linear-elastic, (2) damage, and (3) fracture region. Pure damage-based unloading (dotted, red line) and tension–compression asymmetry are visible. Bottom: exemplary damage onset surface in principal stress space

The proposed material model (Fig. 1, top) consisted of (1) an isotropic, linear-elastic region (initial Young’s modulus $E_{{\mathrm {0}}}$, Poisson’s ratio $\nu$), (2) a nonlinear region where the material degraded based on a scalar damage quantity *D*, and (3) a failure region. The transition from the linear to the nonlinear regime was determined by an isotropic, quadric damage onset surface (adapted from Schwiedrzik et al. (2013), Fig. 1, bottom). It is formulated in terms of the nominal stress tensor $\sigma _{ij}$ as

1 $$\begin{aligned} Y(\sigma _{ij}) = \frac{1}{\mathscr {H}} \left( \sqrt{\sigma _{ij}{\mathscr {F}}_{ijkl}\sigma _{kl}} + F_{ij}\sigma _{ij}\right) - 1 = 0, \end{aligned}$$

with the tensors

2 $$\begin{aligned} F_{ij}&= \frac{1}{2} \left( \frac{1}{\sigma _0^+} - \frac{1}{\sigma _0^-} \right) \delta _{ij}, \end{aligned}$$

3 $$\begin{aligned} {\mathscr {F}}_{ijkl}&= -\frac{\zeta _0}{4} \left( \frac{1}{\sigma _0^+} + \frac{1}{\sigma _0^-} \right) ^2 \delta _{ij}\delta _{kl} \\&\quad + \frac{1 + \zeta _0}{4} \left( \frac{1}{\sigma _0^+} + \frac{1}{\sigma _0^-} \right) ^2 \frac{1}{2}(\delta _{ik}\delta _{jl} + \delta _{il}\delta _{jk}). \end{aligned}$$

Einstein sum convention is used. $\delta _{ij}$ is the Kronecker delta. The shape of the damage surface was defined via a parameter $\zeta _{{\mathrm {0}}}$. It can be adapted to approximate commonly used yield criteria, like Drucker-Prager, von Mises, or Tsai-Wu criterion (Schwiedrzik et al. 2013). The damage onset surface took into account the tension–compression asymmetry of trabecular bone via different tensile and compressive yield stresses ($\sigma _{{\mathrm {0}}}^{{\mathrm {+}}}$, $\sigma _{{\mathrm {0}}}^{{\mathrm {-}}}$). An equivalent formulation in the damage onset strains $\varepsilon _{{\mathrm {0}}}^{\pm }$ was applied. No manual distinction between tension and compression loading was required. Hardening was included via an isotropic hardening modulus $E_{{\mathrm {hard}}}$. The factor ${\mathscr {H}}$ determines the extent of hardening (compare Eq. 1) and depends on the current damage *D*:

4 $$\begin{aligned} {\mathscr {H}} = \frac{1 - E_{{\mathrm {hard}}} / E_{{\mathrm {0}}}}{1 - E_{{\mathrm {hard}}} / (E_{{\mathrm {0}}} (1 - D))}. \end{aligned}$$

### Implementation details

The material degraded locally if the local stress reached the current damage onset surface. In case of material degradation, the modulus was reduced to $E = (1 - D) E_0$. *D* was found numerically by back-projecting the current stress state onto the damage onset surface. Local tissue failure occurred when *D* exceeded a critical value $D_{{\mathrm {c}}}$. Failure was modeled by reducing the modulus to a small residual value $E_{{\mathrm {f}}}$. The material model did not include plasticity or rate dependency. The nonlinear material model was incorporated into ParOSol using a displacement-based, incremental-iterative solving procedure. The details are given in “Appendix C” and Stipsitz et al. (2018). A geometrically linear FE formulation was employed. The FE and material formulation allowed retrospective scaling of the results with the initial modulus $E_{{\mathrm {0}}}$.

### Trabecular biopsies

For objective (2), the extended solver (ParOSolNL) was applied to 20 trabecular bone biopsies. The biopsies were taken from a previous study (Schwiedrzik et al. 2016): the data set consisted of 21 samples from 11 human donors. During experimental testing, 10 samples were loaded until failure in compression and 11 samples were loaded until failure in tension. The samples were cylindrical cores with 8 mm in diameter and 10 mm (tension samples) or 13 mm (compression samples) in height. In this study, one sample was excluded after visual inspection of the microstructure. Segmented $\mu$CT images at a resolution of ${36}\,{\upmu \hbox {m}}$ were available from different locations (9 Femur, 2 Radius, 9 Vertebra). Biopsies from different anatomic sites were included because the aim is a framework that can be applied universally to any trabecular biopsy. An FE mesh was created by converting each voxel of the scans to a linear hexahedral element. Displacement boundary conditions were applied to mimic tension and compression experiments: Nodes on the top plane were displaced in axial direction in strain increments of 0.1%. Nodes on the bottom plane were fully fixed, and all lateral displacements on the top plane were constrained. Analyses were stopped at the first drop in apparent force. Post-ultimate tissue behavior was not suitably modeled due to the lack of large deformation formulation and self-contact constraints.

### Identification of material parameters

The suitable values for the material parameters of the damage model were identified (Table 1). The Poisson’s ratio $\nu$ and $\zeta _0$ were chosen from the literature (Schwiedrzik and Zysset 2015). Following (Schwiedrzik et al. 2013) and using the damage onset strains identified here, $\zeta _0=0.3$ corresponded to an ellipsoidal damage onset surface. A residual stiffness of bone tissue of $E_{{\mathrm {f}}} = 10^{-5} E_0$ was used. The residual modulus has only marginal effects on the results. $E_{{\mathrm {f}}} = 0$ is also possible but may lead to slightly decreased performance. The tissue modulus $E_{{\mathrm {0}}}$ is reported to vary greatly depending on bone type, anatomic location, and age (Carretta et al. 2013). In this study, a homogeneous tissue $E_{{\mathrm {0}}}$ was calculated for each biopsy individually so that the apparent modulus matched the experiment. The remaining parameters ($\varepsilon _0^{\pm }$, $D_{{\mathrm {c}}}$, $E_{{\mathrm {hard}}}$) could not be taken directly from the literature since they depended on the material model and mesh resolution. Instead, the parameters were identified using two biopsy samples, one under compression and one under tension boundary conditions. The parameters were identified by repeatedly performing nonlinear simulations of the two samples. Depending on the simulation results, the parameters were adapted manually to best reproduce the maximum force of the experiments. Only two samples were chosen for the identification process because an optimization routine using all samples would have been computationally demanding and unique results could not be ensured. The results obtained with the identified parameters for all 20 samples justified this practical approach.

**Table 1:** Identified material parameter set for ${36}\,{\mu \hbox {m}}$ mesh resolution

Predefined			Identified on 2 samples				Individual
$\nu$	$\zeta _0$	$E_{{\mathrm {f}}}$	$\varepsilon _0^{+}$ (%)	$\varepsilon _0^{-}$ (%)	$D_{{\mathrm {c}}}$ (–)	$E_{{\mathrm {hard}}}$	$E_{{\mathrm {0}}}$ (GPa)
0.3	0.3	$10^{-5}$	0.68	0.89	0.915	0.05 $E_{{\mathrm {0}}}$	$(7.3-13.5)$

### Post-processing

The resulting apparent stress was defined as the sum of the axial force on the top plane divided by the initial cross-sectional area of the biopsy. Apparent strain was evaluated as the applied displacement divided by the initial height of the cylinders. The apparent yield stress, $\sigma _{{\mathrm {y}}}$, was identified via the 0.2% strain-offset criterion and the maximum stress, $\sigma _{{\mathrm {max}}}$, was the maximum absolute stress in the apparent stress–strain curve. During post-processing, the hexahedral meshes were smoothed for better visualization with ParaView. Additionally, 2D projections of the damage zones were generated to compare the internal fracture patterns. Linear regression analyses were performed for tension and compression samples separately (including the two calibration samples). The intercept of the linear regression function was set to zero. The deviations between simulations and experiments were evaluated for apparent $\sigma _{{\mathrm {y}}}$ and $\sigma _{{\mathrm {max}}}$. The relative errors were obtained by scaling the deviations by the experimental value.

A novel elasticity limit was defined in terms of the percentage of damaged elements ${\mathscr {N}}_{{\mathrm {D}}}$. ${\mathscr {N}}_{{\mathrm {D}}}$ was computed as the number of elements with $(D > 0)$ divided by the total number of elements in the structure. The inelastic start point $\varepsilon _{{\mathrm {ie}}}$ was determined by least-square fitting of the following piece-wise quadratic function $\overline{{\mathscr {N}}}_{{\mathrm {D}}}$ to the individual ${\mathscr {N}}_{{\mathrm {D}}}$ versus total strain curves from simulations (see “Appendix D”):

$$\begin{aligned} \overline{{\mathscr {N}}}_{{\mathrm {D}}}(\varepsilon ) = {\left\{ \begin{array}{ll} 0 &{} \varepsilon \le \varepsilon _{{\mathrm {ie}}} \\ b (\varepsilon - \varepsilon _{{\mathrm {ie}}})^2 &{} \varepsilon \ge \varepsilon _{{\mathrm {ie}}}, \end{array}\right. } \end{aligned}$$

Note that this point is not the apparent 0.2% strain-offset yield point.

**Fig. 2:**
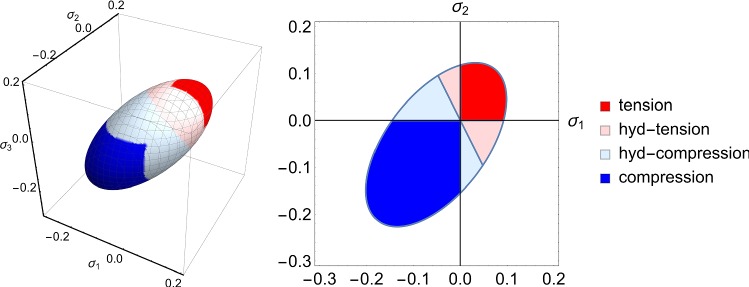
Illustration of the damage mode classification: regions are highlighted on the initial damage onset surface in principal stress space in 3D (left) and on a 2D projection with $\sigma _3 = \sigma _1$ (right). Elements are damaged under local tension (red) or compression (blue) if all principal stress components are positive or negative, respectively. Additionally, hydrostatic damage modes are defined by the sign of the hydrostatic stress: positive hydrostatic stress corresponds to hydrostatic tension (light red) and negative to hydrostatic compression (light blue)

During post-processing, damaged elements were categorized by the stress at initial damage onset (Fig. 2). Compression damage was present if all principal stress components of an element were negative and tension if all principal stress components were positive. The remaining damaged elements were classified by the sign of the hydrostatic stress part (negative corresponded to ‘hydrostatic’ compression, positive to ‘hydrostatic’ tension).

## Results

The simulation time of an individual biopsy sample was between 0.4 and 2 h of real time on a standard shared memory server (2$\,\times \,$14 cores Intel Xeon E5-2697 v2 @2.70GHz, 384 GB RAM). The variation in simulation times was mainly due to the structure sizes; the biopsy models had 3-15 mio DOF. ParOSolNL was very memory efficient; at most 3 GB of total RAM was required.

### Agreement with experiments

**Fig. 3:**
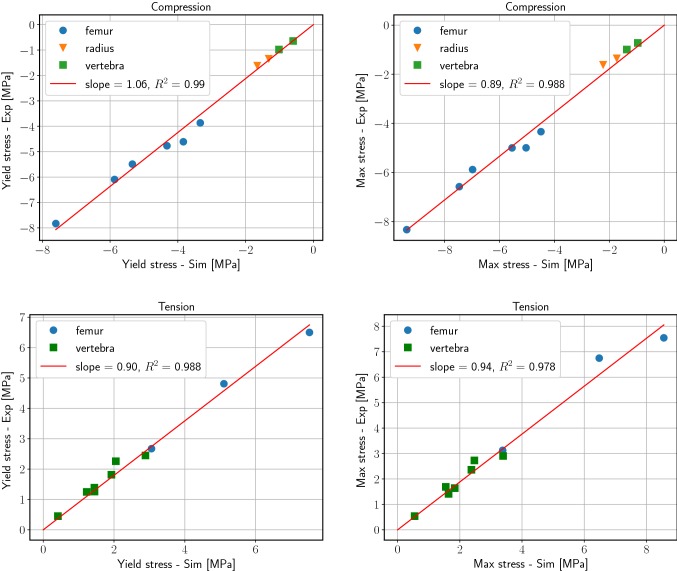
Linear regression analysis for compression (top) and tension (bottom): the apparent 0.2% yield stress (left) and maximum stress (right) obtained in the simulations (on the *x*-axis) are compared to the experimental values (on the *y*-axis, from Schwiedrzik et al. (2016))

The simulation results showed excellent correlation with experiments (Fig. 3). Specifically, apparent 0.2% yield stresses and maximum stresses correlated with a coefficient of determination of $R^2 \ge 0.97$. Maximum stresses were generally overestimated in the simulations (slope of the regression was 0.89 in compression and 0.94 in tension). No correlations in apparent yield strains and ultimate strains were found.

**Fig. 4:**
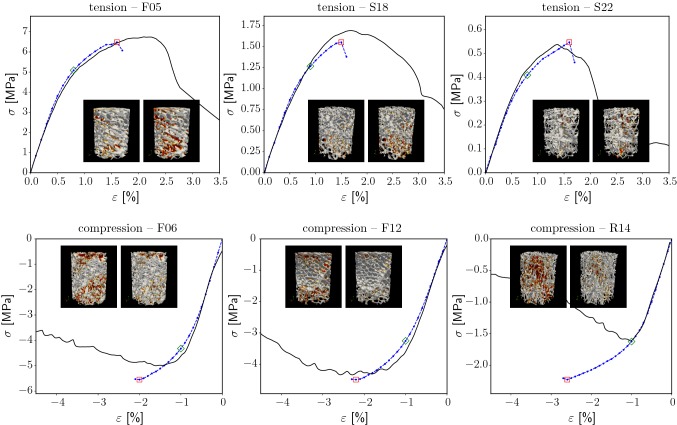
Selected stress–strain curves of experiments (black, solid, from Schwiedrzik et al. (2016)) and simulations (blue, dotted). The green and red rectangles mark the apparent 0.2% yield point and the maximum stress point, respectively. For these two points, the local damage pattern is shown (small pictures: bone structure in gray, damage in red)

**Table 2:** Average relative deviations in the apparent 0.2% yield stress ($\sigma _{{\mathrm {y}}}$) and maximum stress ($\sigma _{{\mathrm {max}}}$) obtained from simulations and experiments (from Schwiedrzik et al. (2016)) for tension and compression samples. Additionally, standard deviations of the relative errors are given

	$\varDelta \sigma _{{\mathrm {y}}} (\%)$	$\varDelta \sigma _{{\mathrm {max}}} (\%)$
Tension	${6.06 \pm 9.39}{\%}$	${4.67 \pm 9.4}{\%}$
Compression	${-5.18 \pm 6.24}{\%}$	${19.65 \pm 12.97}{\%}$

Apparent stress–strain curves showed good qualitative agreement with the experiments (Fig. 4). The local damage patterns differed significantly from sample to sample depending on the individual microstructure (smaller images in Fig. 4). High deviations in apparent $\sigma _{{\mathrm {y}}}$ and $\sigma _{{\mathrm {max}}}$ obtained from simulations and from experiments were observed (Table 2). The maximum stress depended strongly on the critical damage $D_{{\mathrm {c}}}$. In a parameter study using the two calibration samples, ${2}{\%}$ variation in $D_{{\mathrm {c}}}$ led to relative deviations in the maximum stress of approx. ${15}{\%}$. The ${0.2}{\%}$ yield point was nearly unaffected (deviations $< {2}{\%}$). For more details, see “Appendix A”. The influence of the other calibrated material parameters was much smaller; ${10}{\%}$ variation in a parameter resulted in less than ${10}{\%}$ deviations in the apparent yield and maximum point.

### Local damage pattern

**Fig. 5:**
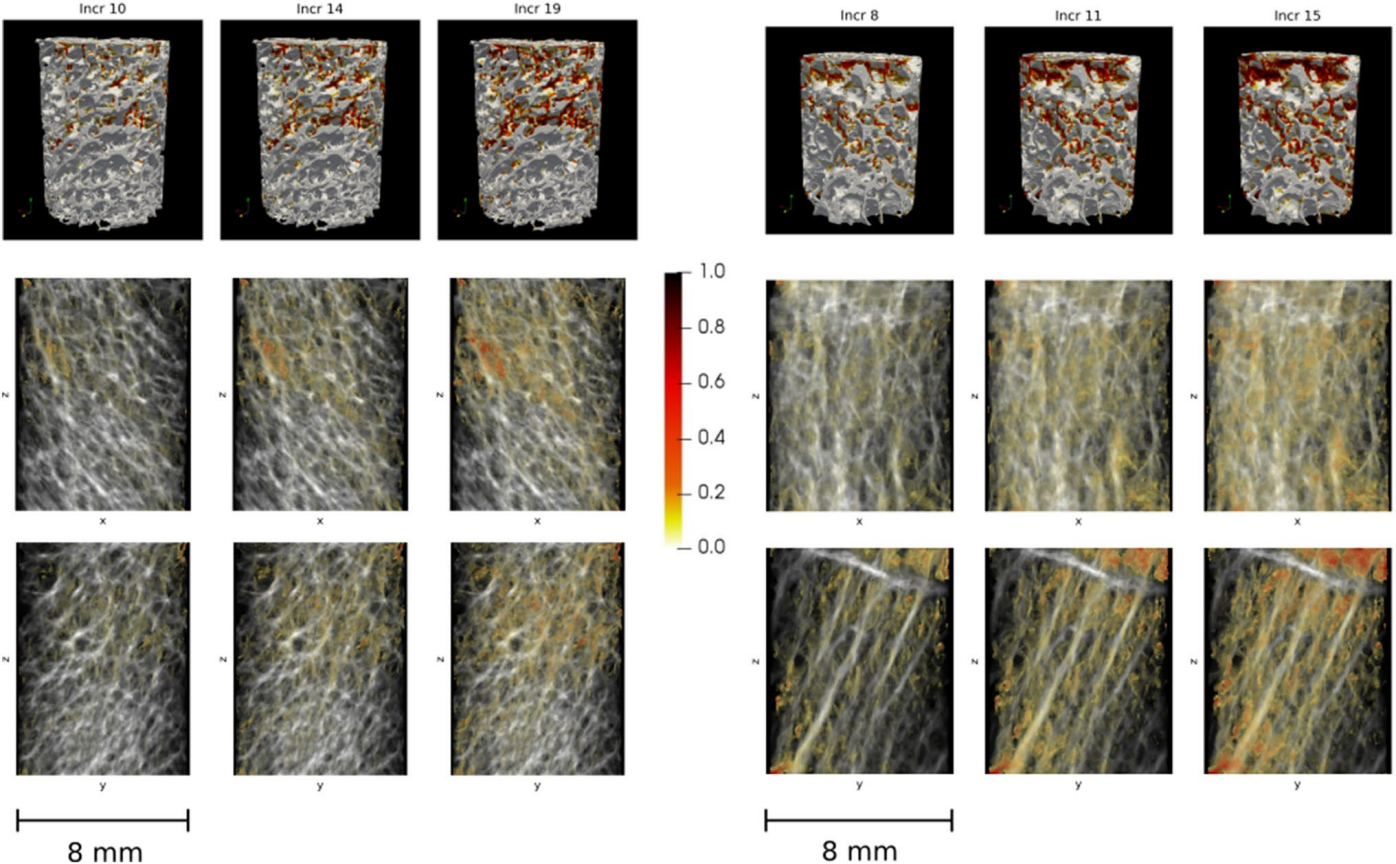
Development of local damage regions over the simulation (pseudo-) time. One sample under compression (left) and one under tension (right) are shown. The first columns give the local damage at the apparent 0.2% yield point and the third columns at the maximum point. The structures are shown in 3D (top row) and in two perpendicular projections (middle and bottom row). The bone structure is depicted in grayscales, the damage *D* in red

The local development of damage regions was observable due to the nonlinear nature of the simulations. Damage patterns differed depending on the individual microstructures. Two selected local results are given in Fig. 5. At the ${0.2}{\%}$ yield point (left columns), already some sizable damage regions existed. As the applied load increased, initially damaged regions degraded further and damage regions grew. The maximum sustainable load was reached in the right columns of Fig. 5. In most samples, a diffuse damage pattern dominated.

**Fig. 6:**
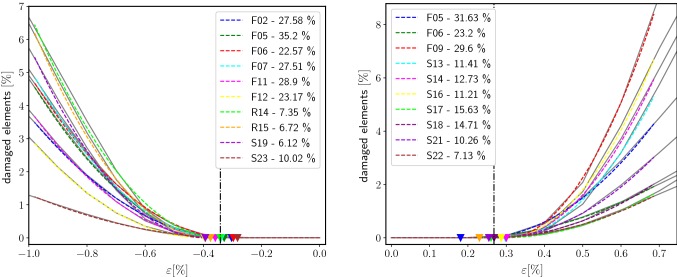
The percentage of elements that are damaged ($D > 0$) shows a sudden increase in the inelastic start point $\varepsilon _{{\mathrm {ie}}}$. The colored triangles denote $\varepsilon _{{\mathrm {ie}}}$ of the individual samples and the dotted line marks the average $\varepsilon _{{\mathrm {ie}}}$. The inelastic region starts later for compression (left) than for tension boundary conditions (right)

The local damage allowed the definition of a novel elasticity limit directly from simulation results. The inelastic start point $\varepsilon _{{\mathrm {ie}}}$ was defined as the strain where a pronounced nonlinearity occurred, manifesting in an increase in damaged elements ${\mathscr {N}}_{{\mathrm {D}}}$ (Fig. 6). The individually fitted curves $\overline{{\mathscr {N}}}_{{\mathrm {D}}}$ and inelastic start points $\varepsilon _{{\mathrm {ie}}}$ are depicted in Fig. 6. On average, $\varepsilon _{{\mathrm {ie}}}$ was $0.27 \pm {0.04}{\%}$ for tension and $-0.34 \pm {0.04}{\%}$ for compression samples. No systematic difference between low- and high-density samples was found. For comparison, the apparent ${0.2}{\%}$ yield strain was determined as $0.67 \pm 0.16\%$ in tension and $-0.81 \pm 0.13\%$ in compression. The ${0.02}{\%}$ yield strain was $0.26 \pm 0.18\%$ in tension and $-0.2 \pm 0.17\%$ in compression.

**Fig. 7:**
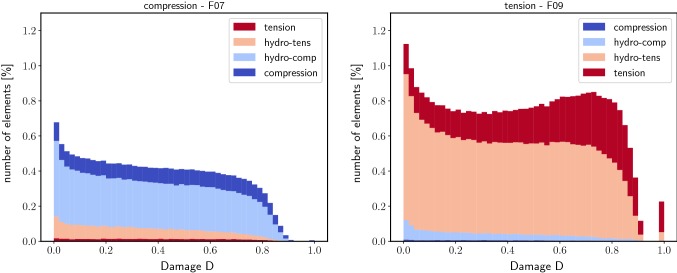
Damage distribution of one femur biopsy under compression (the corresponding local damage pattern is shown in Fig. 5, left) and one femur biopsy under tension (local damage pattern see Fig. 5, right) at the maximum stress point. Contributions of the different initial damage modes are shown

Damage distributions revealed qualitative differences between the tension and the compression group (Fig. 7). At the maximum stress point, samples under tension showed a peak which was not present in damage distributions of compression samples. At this point, in samples under apparent compression, a larger amount of tension damage was present than vice versa ($2.61 \pm 0.71 \%$ compared to $0.12 \pm 0.06 \%$).

## Discussion

The aims of this study were (1) the development of an efficient, materially nonlinear $\mu \hbox {FE}$ solver including tissue-level failure and (2) the investigation of its potential based on trabecular bone biopsies. A simple, damage-based material model was successfully incorporated in ParOSol while preserving its good performance. Material parameters were identified and resulted in excellent correlation between the maximum stress in simulations and experiments. The development of local damage regions was observable due to the nonlinear nature of the simulations. This led to the definition of a new elasticity limit based on the evolution of the number of damaged elements. Damage distributions allowed more insight into the internal processes of the trabecular bone biopsies.

Regarding objective (1), the excellent parallel performance of the original solver was successfully ported to the nonlinear material regime. Simulation times using ParOSolNL were 10 times lower compared to ParFEAP (Schwiedrzik et al. 2016). For the same samples and on the same machine, they reported solving times between 4 and 22 hours. While many solvers can simulate models with a couple of mio DOF, ParOSolNL has already been successfully applied to huge bone structures as well. For details on the performance, the reader is referred to Stipsitz et al. (2018). To summarize, bone simulations of more than 5 billion DOF were feasible on a standard HPC cluster. ParOSolNL used the computational resources efficiently and scaled well with at least 1024 CPUs, while maintaining a low memory footprint. No convergence issues were encountered for any sample. The geometric multigrid preconditioner used in solving the linear equations is robust against modulus jumps (Flaig 2012). Thus, setting $E = 0$ in failed elements does not deteriorate the performance. For solving the nonlinear problem, an incremental-adaptive procedure was used. Since the material formulation is not continuous at tissue failure, damage was not allowed to decrease if it exceeded the critical damage in any iteration.

The material parameters identified for objective (2) compare well to the parameters used in the literature (Table 3). A range of tissue moduli is reported here, since $E_{{\mathrm {0}}}$ was identified for each biopsy individually. The tensile yield strain is in the range of values reported in the literature. The tension–compression asymmetry in the tissue yield strains is slightly higher. The value for the hardening modulus matches the one reported in Bayraktar et al. (2004). The material parameters were calibrated on two samples only. Although they led to good agreement of simulation results with experiments, it cannot be assumed that the parameters are generally valid or the best possible parameters. However, the goal of this work was not the identification of physical tissue properties but the development of a framework that can be universally applied to any trabecular biopsy. It is assumed that on the tissue level, i.e., at around ${30}\,{\upmu \hbox {m}}$ resolution, the tissue properties are the same irrespective of anatomic site. To account for stiffness variations, the results are scaled to match the experimental stiffness. The variations in the stiffness could be caused among others by the degree of mineralization or errors in representing the exact boundary conditions from experiments.

**Table 3:** Trabecular tissue properties reported in the literature and identified in this study: initial tissue modulus $E_{{\mathrm {0}}}$, initial tensile yield strain, yield strain asymmetry, and hardening modulus $E_{\text{hard}}$

Property	Literature	Reference(s)	This study
$E_0$ (GPa)	1–15	Lucchinetti et al. (2000)	7.3–13.5
$\varepsilon _{{\mathrm {0}}}^{{\mathrm {+}}}$ (%)	0.4–2.6	Frank et al. (2018)	0.68
		Carretta et al. (2013)	
$\varepsilon _{{\mathrm {0}}}^{{\mathrm {+}}} / \varepsilon _{{\mathrm {0}}}^{{\mathrm {-}}}$	0.4, 2 / 3	Schwiedrzik et al. (2016)	0.76
	0.62	Bayraktar et al. (2004)	
$E_{{\mathrm {hard}}}[E_{{\mathrm {0}}}]$	0.05	Bayraktar et al. (2004)	0.05

Simulation results showed excellent correlation with experiments. The apparent ${0.2}{\%}$ yield stress and ultimate stress fit well to the experiments for a wide range of different trabecular structures (different anatomic locations, bone densities) under axial tension and compression. This high correlation with the experiments confirms the simple material identification procedure. Good correlation in apparent-level yield stress is generally reported in the literature for nonlinear $\mu \hbox {FE}$ simulations (Schwiedrzik et al. 2016; Sanyal et al. 2012; Hambli 2013). However, most $\mu \hbox {FE}$ material models do not include tissue fracture. In one of the few exceptions, a comparable correlation for the maximum stress is reported (Hambli 2013). However, their solver was exclusively applied to small biopsies.

The maximum stress found in the simulations is very sensitive to small variations in the critical damage $D_{{\mathrm {c}}}$. Different values for $D_{{\mathrm {c}}}$ in tension and compression have been found to improve the results (see “Appendix A”). However, in each global loading condition, a mixture of different local loading conditions occurred. Thus, different global values for $D_{{\mathrm {c}}}$ for tension and compression samples are not consistently possible. In the material model, different values for $D_{{\mathrm {c}}}$ for local tensile or compressive loading would require a tensor formulation for the damage to account for mixed loading cases.

The ultimate strength of low-density samples under compression is systematically overestimated (a representative stress–strain curve is compression—R14 in Fig. 4). Slender structures under compression, which are common in low-density samples, are liable to buckling and extensive bending (Cowin 2001; Stölken and Kinney 2003). However, in this study, a linear geometric FE formulation is applied, which cannot reproduce these mechanisms. Thus, slender trabeculae seem to withstand much higher strains than physically possible, leading to an overestimation of the overall strength.

The location of damaged regions obtained in the simulations looks plausible. However, further experiments are required to validate the location of failure and to study the reliability of local results. Rather diffuse, non-localized damage was visible up to the ultimate point where a more localized failure occurred. Local information is not easily obtained in experiments, especially during loading (Carretta et al. 2013). Mostly, the crack pattern is studied by staining the structure (Moore and Gibson 2002) or by simultaneous $\mu$CT scanning (Thurner et al. 2006). With the recent developments in digital volume correlation, a direct local comparison between displacements in experiments and simulations could become possible soon (Costa et al. 2017).

A considerable amount of elements were damaged already very early in the simulations. At the apparent ${0.2}{\%}$ yield point on average, (2–6)% of the elements were damaged. Thus, the novel elasticity limit determined directly from the simulations was lower than the apparent ${0.2}{\%}$ yield point. The $\varepsilon _{{\mathrm {ie}}}$ fits well with the physiologic strains reported in the literature (0.05–0.6%) (Yang et al. 2011; Di Palma et al. 2003). The elasticity limit reflected the tension–compression asymmetry found in bone due to the asymmetric tensile and compressive damage onset strains.

As expected, simulation results showed no systematic differences between low- and high-density samples. This agrees well with the assumption that strain at fracture is comparable in different bones (apart from the tension–compression asymmetry) while fracture stress can vary largely (Morgan and Keaveny 2001).

Damage distributions suggested systematic differences in the damage mechanism between external tension or compression loads. Under applied compression, a larger amount of tension damage was present than vice versa. One reason could be the higher compression than tension tissue yield strain. Additionally, it could indicate different predominant loading modes under tension and compression. Compression samples show mainly compression damage but also higher amounts of tension damage. This could be due to a mixture of local compression and bending. A similar bending behavior has been reported in Harrison et al. (2013) and Shi et al. (2010).

The good performance comes with a number of limitations due to the simple modeling approach: First, static analyses were performed which included material nonlinearity only. No large deformation or contact mechanisms were applied. It is well known that a linear geometric formulation may lead to decisive errors in samples with a bone volume density of less than ${20}{\%}$ (Bevill et al. 2006). This is in accordance with the high deviations found in this study for low-density samples under compression. In the future, it needs to be reconsidered if an extension to overcome geometric nonlinearity is possible without deteriorating the good performance. Second, the material model did not include plasticity and strain rate dependency. Third, the results are mesh dependent (see “Appendix B”). Thus, the material parameters identified here are only valid for the mesh resolution of ${36}\,{\mu\hbox{m}}$. Three aspects concerning mesh accuracy have to be discussed: (1) Meshing a structure with aligned hexahedral elements leads to ragged surfaces. Thus, the results, especially stresses, oscillate on curved surfaces (Guldberg et al. 1998). However, hexahedral elements enable an efficient and highly parallel HPC implementation with a low memory footprint. (2) Local continuum damage is known to be strongly mesh size dependent since a strong localization of damage occurs. However, in this case, the diffuse damage behavior opposes this effect. It was found that the damage does not localize to single elements. Instead, larger damage regions form. (3) The nonlinearity of the system makes the results strongly dependent on small structural deviations as introduced by coarsening the structure. Thus, local results should be viewed with caution. In the future, a local validation study has to be performed to check the local accuracy of the chosen approach. The simple material model and FE formulation were chosen because the main focus of this work was the development of a fast and efficient solver which can be readily applied to large-scale biomechanical problems.

### Conclusion

Although a very simple material model and algorithm were used, quite good agreement between simulations and experiments was achieved. The new framework, ParOSolNL, enables nonlinear simulations of large structures with suitably high resolution in reasonable simulation times. The development of damage regions can be traced in detail due to the nonlinear nature of the simulations. Additionally, a new elasticity limit is proposed which requires only information obtained directly from the simulations. Interesting differences in damage distributions between tension and compression were found. Further investigations of these differences in the course of future nonlinear applications, for instance on whole bones, may help to gain more insight into the internal mechanisms of bone failure.

## Figures and Tables

**Fig. 8:**
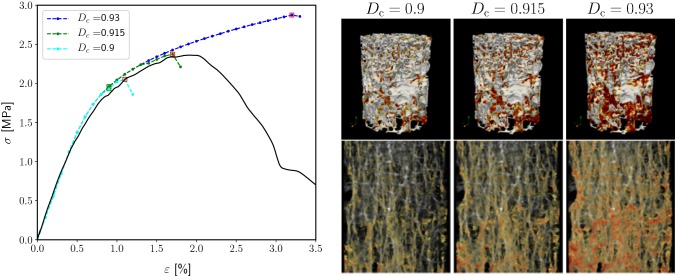
The maximum stress point depends strongly on slight variations (here: 2%) in the critical damage $D_{{\mathrm {c}}}$ (left). The results for one vertebra biopsy under tension are shown. A higher $D_{{\mathrm {c}}}$ corresponds to more damaged elements at apparent failure (right)

**Fig. 9:**
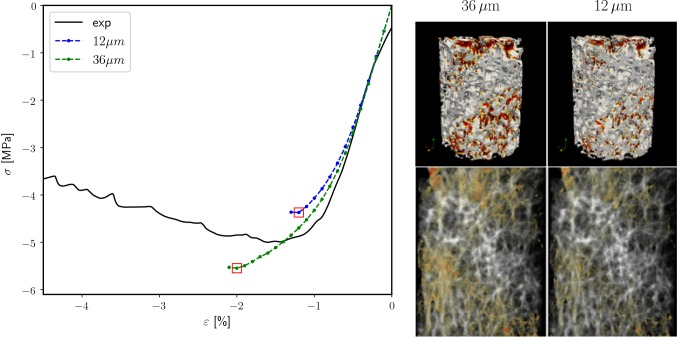
Dependency of the simulation results on the mesh resolution: apparent stress–strain curve for one biopsy from a femur ($\text {BV}/\text {TV} = 22.57\%$) under compression with ${36}\,{\upmu \hbox {m}}$ and ${12}\,{\upmu \hbox {m}}$ resolution (left) and corresponding local results (middle and right) at the maximum stress point

**Fig. 10:**
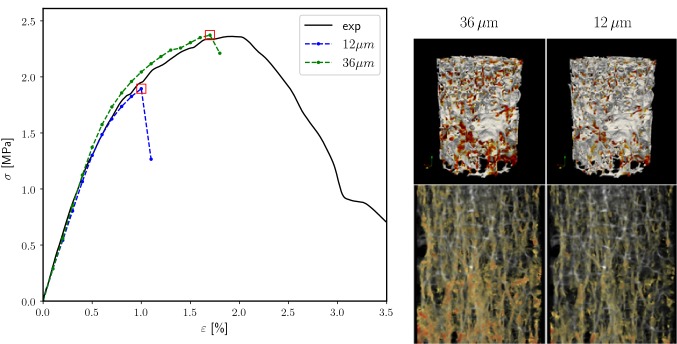
Dependency of the simulation results on the mesh resolution: apparent stress–strain curve for one biopsy from a vertebra ($\text {BV}/\text {TV} = 11.41\%$) under tension with ${36}\,{\upmu \hbox {m}}$ and ${12}\,{\upmu \hbox {m}}$ resolution (left) and corresponding local results (middle and right) at the maximum stress point

**Fig. 11:**
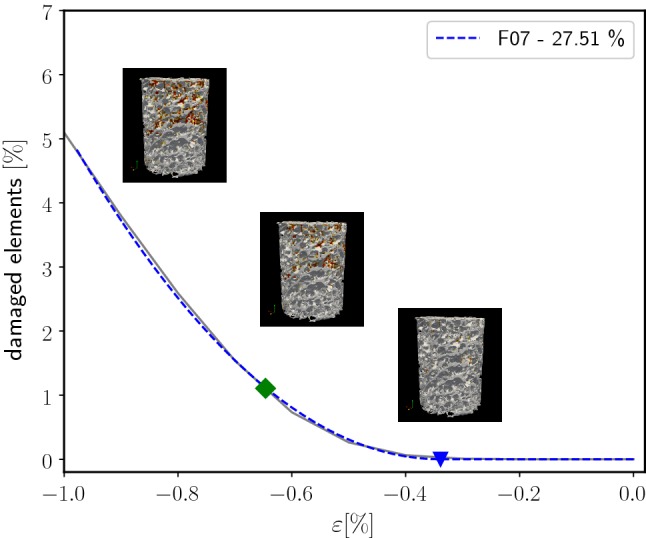
Evaluation of the elasticity limit for one individual sample under compression: the number of damaged elements is defined as the sum of all elements with $(D>0)$ divided by the total number of elements in the structure. A piece-wise quadratic function is fitted to the initial part of the curve. The obtained elasticity limit (blue triangle) is usually lower than the 0.2% yield point (green rectangle). At the 0.2% yield point, already a sizeable amount of damage is present (see small images)

## Appendix A: Implementation details

The nonlinear solver is an extension of the open-source solver ParOSol (Flaig, 2012) which is able to solve linear-elastic FE problems in a highly parallel and efficient way. Additionally, it has a very low memory footprint which is important for the application on standard high-performance clusters.

An incremental-iterative procedure was applied to include nonlinear material behavior into ParOSol. At the start of each increment, an explicit step was performed to obtain a start value for the damage of each element at the current loading state. The initial solution was then iteratively corrected until a converged solution was found. During these iterations, damage was allowed to decrease compared to the initial solution, but not below the solution of the previous increment. To assure convergence, elements that were once fractured ($D > D_{{\mathrm {c}}}$) remained fractured in all succeeding iterations.

The actual solving of the FE equations was performed by the linear solver within the original ParOSol. In each iteration (of each increment), the Young’s moduli in the structure were kept constant. The resulting linear system of equations was solved using the linear solver of the original ParOSol. For the solving process, fully integrated linear hexahedral elements were applied. For the evaluation of the damage onset criterion, the interpolated centroid stress of each element was used. The modulus was reduced element-by-element since this best fitted the design of the original ParOSol.

The solution of an increment was taken to be sufficiently converged if the change in damage was small enough. Two convergence criteria were defined: (1) The local change in damage, $^{{{\langle {\mathrm {e}} \rangle }}}R_{{\mathrm {1}}}$, from iteration $(i-1)$ to iteration (*i*) was defined as

5 $$\begin{aligned} ^{{{\langle {\mathrm {e}} \rangle }}}R_{{\mathrm {1}}} = ^{{{\langle {\mathrm {e}} \rangle }}}\!\!D_{{n}}^{{(i)}} - ^{{{\langle {\mathrm {e}} \rangle }}}\!\!D_{{n}}^{{(i-1)}} \le 100 \delta . \end{aligned}$$

(2) The average change in damage, $R_{{\mathrm {2}}}$, was

6 $$\begin{aligned} R_{{\mathrm {2}}} = \frac{\sum _{\mathrm {all\ elements}} {^{{{\langle {\mathrm {e}} \rangle }}}R_{{\mathrm {1}}}}}{N} < \delta . \end{aligned}$$

$\delta$ is the convergence tolerance, and $\sum _{\mathrm {all\ elements}}$ denotes the sum over all elements. *N* is the number of elements for which the damage has changed from iteration $(i-1)$ to iteration (*i*).

## Appendix B: $D_{{\mathrm {c}}}$ sensitivity

The maximum stress found in the simulations depended strongly on the choice of $D_{{\mathrm {c}}}$ (Fig. 8). The lower $D_{{\mathrm {c}}}$ was chosen, the earlier global failure occurred. However, the slope of the stress–strain curve was only changed minimally and primarily in the vicinity of the ultimate point. A lower value for $D_{{\mathrm {c}}}$ led to a smaller number of damaged elements at apparent fracture (local images in Fig. 8, right). The material degradation started in the same regions, independently of $D_{{\mathrm {c}}}$.

The strong influence of $D_{{\mathrm {c}}}$ was one reason for the poor prediction of the maximum stress values in compression samples. The maximum stress was systematically overestimated for the compression biopsies, especially in low-density samples. Decreasing $D_{{\mathrm {c}}}$ by ${2}{\%}$ and re-simulating all compression samples led to reduced errors in the maximum stress (Table 4). The slope of the linear regression curve for the maximum stress reached nearly unity (1.02), while the coefficient of determination was not affected.

**Table 4:** Average relative deviations in the apparent ${0.2}{\%}$ yield stress ($\sigma _{{\mathrm {y}}}$) and maximum stress ($\sigma _{{\mathrm {max}}}$) obtained from simulations with ${2}{\%}$ reduced $D_{{\mathrm {c}}}$

	$\varDelta \sigma _{{\mathrm {y}}} (\%)$	$\varDelta \sigma _{{{\max }}}$ (%)
Compression (reduced $D_{{\mathrm {c}}}$)	${-5.65 \pm 6.3}{\%}$	${4.35 \pm 10.62}{\%}$

## Appendix C: Mesh resolution

Simulation results depended strongly on the mesh resolution. The mesh dependency stems from the purely local damage formulation of the material model. In Figs. 9 and 10, the influence of the mesh resolution can be observed. The amount of damaged elements decreased with lower voxel size. However, the location of maximum damage and fracture in the trabecular structures remained approximately the same. As expected, the width of the failed regions was much thinner when the bone structure was finer resolved. Global results were strongly affected by the mesh resolution. Thus, the identified material parameters are only suitable for a resolution of ${36}\,{\upmu \hbox {m}}$ for which they were identified.

## Appendix D: Elasticity limit

For the definition of an elasticity limit, the evolution of damage over the applied strain is used (Fig. 11).
